# Port Placement Strategies for Robotic Pulmonary Lobectomy: A Narrative Review

**DOI:** 10.3390/jcm11092612

**Published:** 2022-05-06

**Authors:** Sara Parini, Fabio Massera, Esther Papalia, Guido Baietto, Giulia Bora, Ottavio Rena

**Affiliations:** 1 Division of Thoracic Surgery, Ospedale Maggiore della Carità, 28100 Novara, Italy; fabiomassera@icloud.com (F.M.); esther.papalia@libero.it (E.P.); g.baietto@virgilio.it (G.B.); giulia.bora@libero.it (G.B.); ottavio.rena@uniupo.it (O.R.); 2 Department of Health Sciences, Università del Piemonte Orientale, 28100 Novara, Italy

**Keywords:** RATS, robotic surgery, robotic lobectomy, surgical technique, robotic approach, port mapping

## Abstract

Background: Despite the use of robotics becoming increasingly popular among thoracic surgeons worldwide, there remains debate over the best robotic approach for lung resections. In this paper, we delineated the main port placement strategies and discussed their advantages and disadvantages. Methods: A PubMed literature review was performed using key phrases such as “robotic lobectomy technique”, “RATS lobectomy”, and “port placement robotic lobectomy”. After the final review, 22 articles were included as references, of which 10 described common robotic port mapping techniques. Results: Several port strategies for robot-assisted pulmonary lobectomies have been proposed and described in the literature, each showing its own limitations and advantages. Conclusions: New robotic surgeons may choose their port strategy according to personal preference and previous surgical experience, especially regarding open or VATS resections. Robust data comparing different port placements in robotic surgery are lacking. Further research should be directed toward comparisons of clinical outcomes with different robotic approaches.

## 1. Introduction

Robotic surgery has several advantages, including three-dimensional binocular vision, elimination of the natural surgeon tremor, an increased degree of motion, and improved dexterity over VATS. Limitations of robotic surgery include high initial capital costs, longer setup time, lack of ability to palpate the lung, and lack of haptic feedback [1,2]. Since the first report of robot-assisted lobectomy, the operation has evolved into a wide range of approaches. Each of them has several variations described in the literature. The robotic system itself has deeply evolved over the years. In the early years of robotic thoracic surgery, the approach was similar to VATS. In fact, the first port placement strategies were developed using the initial robot platform (da Vinci S/Si). Starting from 2016, the introduction of a more versatile version of the robotic system (da Vinci Xi) led to an evolution of port mapping as well [2,3,4].

Despite the use of robotics becoming increasingly popular among thoracic surgeons worldwide, there remains debate over the best robotic approach for lung resections. In this paper, we delineated the main port placement strategies and discussed their advantages and disadvantages. As there is no one agreed-upon way to conduct the procedure, the aim of the paper is to provide guidance to surgeons eager to navigate the pathway of robotic training and the different port placement options.

## 2. Materials and Methods

**Figure 1:**
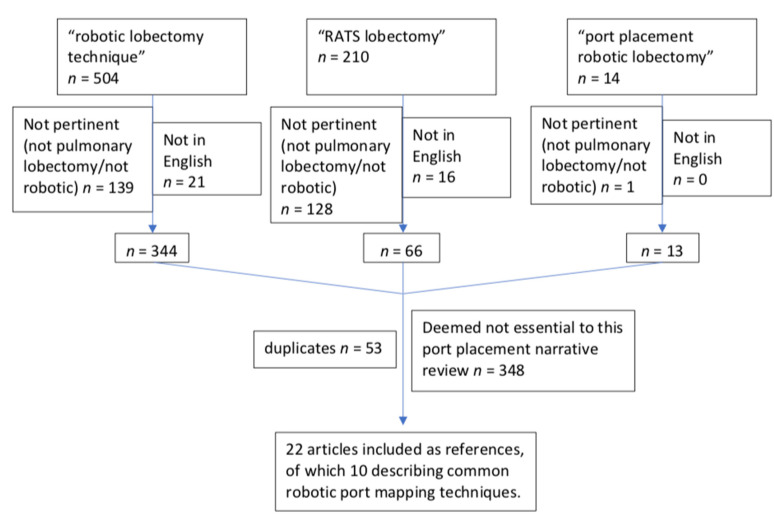
Flow chart summarizing the selection of included articles.

A PubMed literature review was performed using key phrases such as “robotic lobectomy technique” (504 results), “RATS lobectomy” (210 results), and “port placement robotic lobectomy” (14 results). This search was performed in February 2022, capturing all papers published prior to this date. Inclusion criteria were studies written in English describing robot port mapping techniques for pulmonary resections. Articles were reviewed by the authors (S.P., O.R.) to determine relevance, and references were reviewed in order to expand the relevant data collection. After the final review, a total of 22 articles were identified as pertinent to this review and included in the references, of which 10 were included in the main table as they described common robotic port mapping techniques. This search has been summarized in Figure 1.

## 3. Results and Discussion

Table 1 describes the potential advantages and disadvantages of the main robotic port placement strategies. In broad terms, two main options can be adopted. The first is a robotic-assisted approach; it combines a utility incision (similar to the one used in VATS) with robotic ports [3], the pleural cavity is in direct communication with the environment, and CO_2_ is not insufflated into the chest. The second is a total port approach; the incisions are as large as the size of the trocars placed in them, and CO_2_ is insufflated into the closed chest.

### 3.1. Robot-Assisted Thoracic Surgery (RATS)

**Figure 2:**
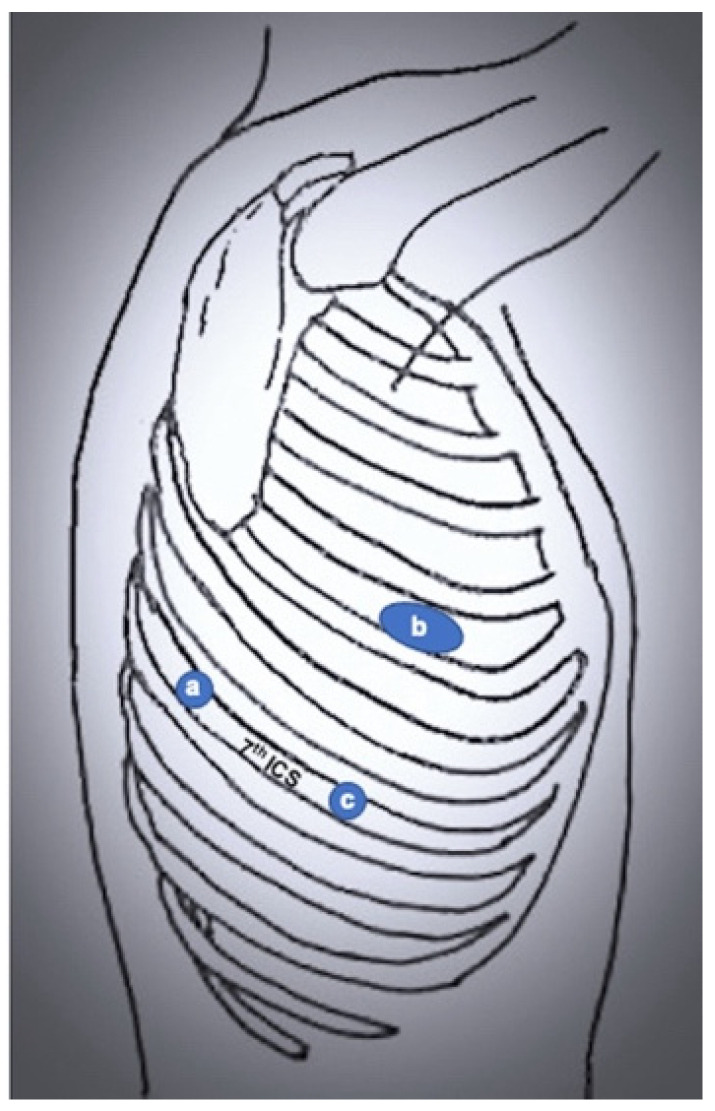
Port mapping of 3-port RATS. (**a**) robotic left arm; (**b**) utility incision; (**c**) camera port.

**Figure 3:**
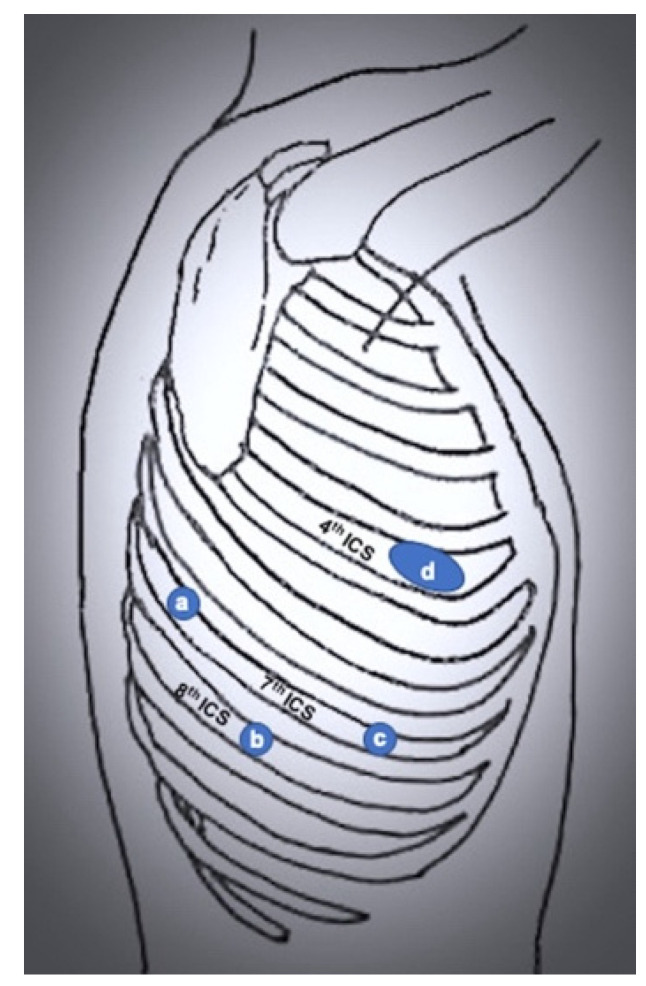
Port mapping of 4-port RATS. (**a**) robotic left arm 1 (lung retraction); (**b**) robotic left arm 2; (**c**) camera port; (**d**) utility port.

The first robot-assisted approach was described by Melfi et al. [3], who reported the first series of pulmonary lobectomies performed using robotic surgery in the early 2000s. This technique was developed when the robotic system was the Da Vinci standard. It consists of a three-arms approach with an additional incision for the assistant surgeon. Specifically, a 3 cm utility port is placed in the 4th–5th intercostal space with the right robotic arm, a camera port in the 7th–8th intercostal space midaxillary line, a robotic left port in the 6th–7th intercostal space posterior axillary line, and the assistant port between utility and camera port. This approach was later adopted and modified by Park et al. [5] and Veronesi et al. [6]. Park and colleagues used conventional thoracoscopy incision (at the level of the superior vein midaxillary line for upper lobectomies, one intercostal space lower for middle and lower lobectomies), a camera port in the 7th–8th intercostal space posterior axillary line, and another incision above the diaphragm posterior to the tip of the scapula (Figure 2). Veronesi and colleagues maintained four robotic ports, resulting in a 3 cm utility thoracotomy in the 4th intercostal space midaxillary line, a camera port in the 7th intercostal space midaxillary line, a robotic port in the 8th intercostal space posterior axillary line, and a robotic port 7th intercostal space behind the tip of the scapula (used for lung retraction) (Figure 3).

This port mapping has the main advantage of providing an access incision of 3–4 cm, making this approach similar to VATS. For this reason, it may be the easiest way to start for surgeons with previous VATS experience. However, the first surgeon must rely on the bedside assistant, who is frequently a trainee, for many dangerous steps such as stapling the vessels. On the other hand, its combined nature limits many advantages of complete portal robotic surgery, as CO_2_ cannot be insufflated inside the cavity, thus providing only limited working space and visibility. Moreover, despite a utility incision similar to the VATS approach, the total number of incisions is higher than the number of incisions in VATS.

**Figure 4:**
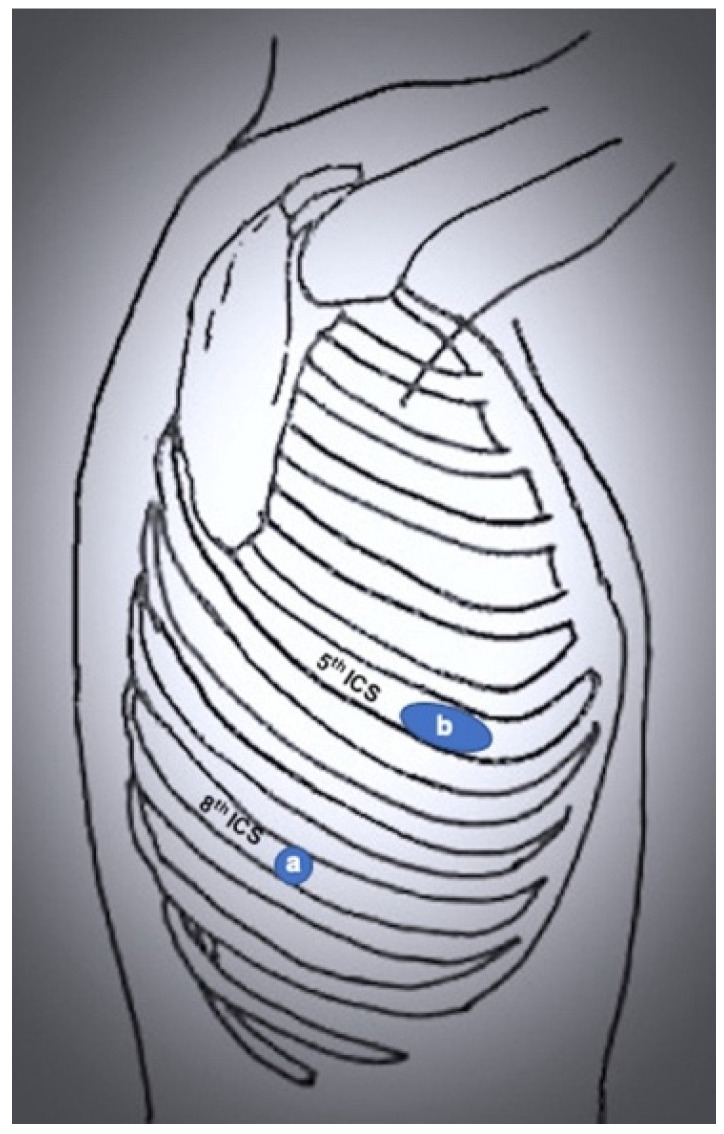
Port mapping of biportal RATS. (**a**) robotic left arm; (**b**) utility and camera port.

Recently, a biportal RATS approach has been described by Yang and colleagues [7], with an 8 mm incision in the 8th intercostal space posterior axillary line and a 4 cm incision in the 6th intercostal space anterior axillary line. A hook is on the right arm, and robotic forceps are on the left. The hilar structures are divided with a stapler by the assistant through the utility port (Figure 4).

According to Yang et al. [8], a uniportal RATS can be performed through a single, 4 cm incision in the 4th intercostal space midaxillary line. A 30° camera is placed at the upper end of the incision, and two other robotic arms (forceps and hook) are placed in the upper two-thirds of the incision. At the lower end, a 1.5 cm space is left for the assistant to help.

This technique may shorten the docking time and combine the advantages of RATS and biportal/uniportal VATS. However, the uniportal access may provide limited maneuverability as the two robotic instruments are crossed inside the thorax. The lung retractor, staplers, clips, and suction need to be introduced by a bedside assistant with previous experience in uniportal VATS.

Recently, some groups reported surgical cases performed using a subxiphoid utility port [9,10]. In this case, four intercostal ports are created above the ninth rib, and a subxiphoid utility port is added. The pleural cavity is reached by blunt dissection. This approach may reduce the clashing between instruments, offers a good angle for stapling, and enables specimen removal.

### 3.2. Completely Portal Robotic Lobectomy (CPRL)

The evolution of the robotic system led to the development of new port placement techniques and to the possibility to perform a completely portal robotic lobectomy. Three and four robotic arms can be used [11]. Compared with robot-assisted techniques, a completely port-based approach offers the advantage of a closed environment, with more working room due to the insufflation of CO_2_, and it allows for partial dissection of gas into the local tissue planes. The extraction port, which enlarges a trocar incision at the end of the operation, does not preclude CO_2_ insufflation [1]. On the other hand, the insufflation of CO_2_ leads to a rise in PaCO_2_, which has been shown to decrease pulmonary compliance. It can also increase airway pressures and decrease tidal volumes resulting in hypercarbia [12,13]. Other disadvantages of this approach include the fact that it does not allow the surgeon to palpate the lung, and that specimen extraction often requires the enlargement of the incision.

**Figure 5:**
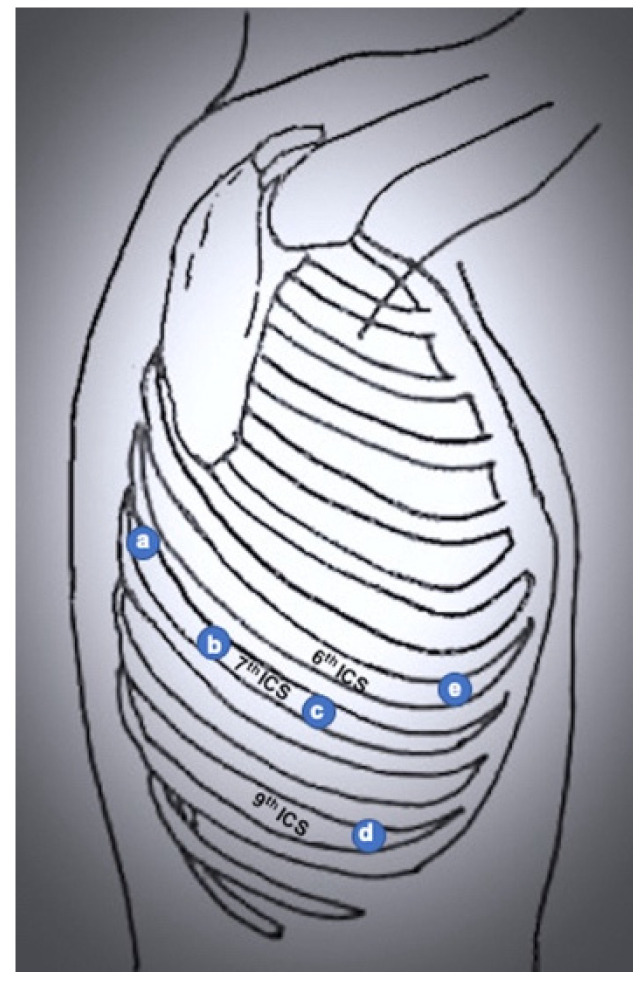
Port mapping of 4-port completely portal robotic lobectomy. (**a**) Robotic left arm 1 (lung retraction); (**b**) robotic left arm 2; (**c**) camera port; (**d**) assistant port; (**e**) robotic right arm.

In 2011, Cerfolio and colleagues [1,14,15,16] described a completely portal robotic lobectomy, using four robotic arms with an assistant port. The pleural space is entered using a port in the midaxillary line over the top of the seventh rib. The most posterior port is made of at least two intercostal spaces below the major fissure (usually the 7th), and the other ports are placed in the same intercostal space, at a minimum distance of 9 cm between each other (Figure 5). The same author [15] later applied a more caudal port positioning (over the top of the 8th or 9th rib), with the most posterior port two ribs below the major fissure just anterior to the spinal process of the vertebral body, another port 10 cm anteriorly, and the last two incisions made with a 9 cm distance from each other and a utility incision between the two, in the 9th intercostal space.

Another version of CPRL consists of a four-arms approach without an assistant port; in this case, the camera port is positioned in the 7th–8th intercostal space posterior axillary line, the posterior ports are placed along the same intercostal space, the anterior port is just above the diaphragm (or 4th–5th intercostal space, similar to the robot-assisted approach) anterior axillary line [4,17,18].

Ninan and Dywleski [19] described a completely portal robotic lobectomy using three arms, with an assistant port at the tip of the 11th rib (the chest is entered through the 8th intercostal space). The robotic camera port is placed in the 5th–6th intercostal space over the mid-fissure area. Two other ports are placed in the same intercostal space anteriorly and posteriorly.

As there is not a universal port strategy for robot-assisted lobectomy, Oh et al. [20] surveyed 100 high volume thoracic surgeons in the US and found that 90% of them utilized a 4-arm approach and 79% used a completely 4-arm portal approach with CO_2_ insufflation. Ten percent utilized a 3-arm technique, and of these, 50% utilized a completely portal approach. The use of multiple different interspace levels was common, and most surgeons used an additional nonrobotic assistant port.

**Figure 6:**
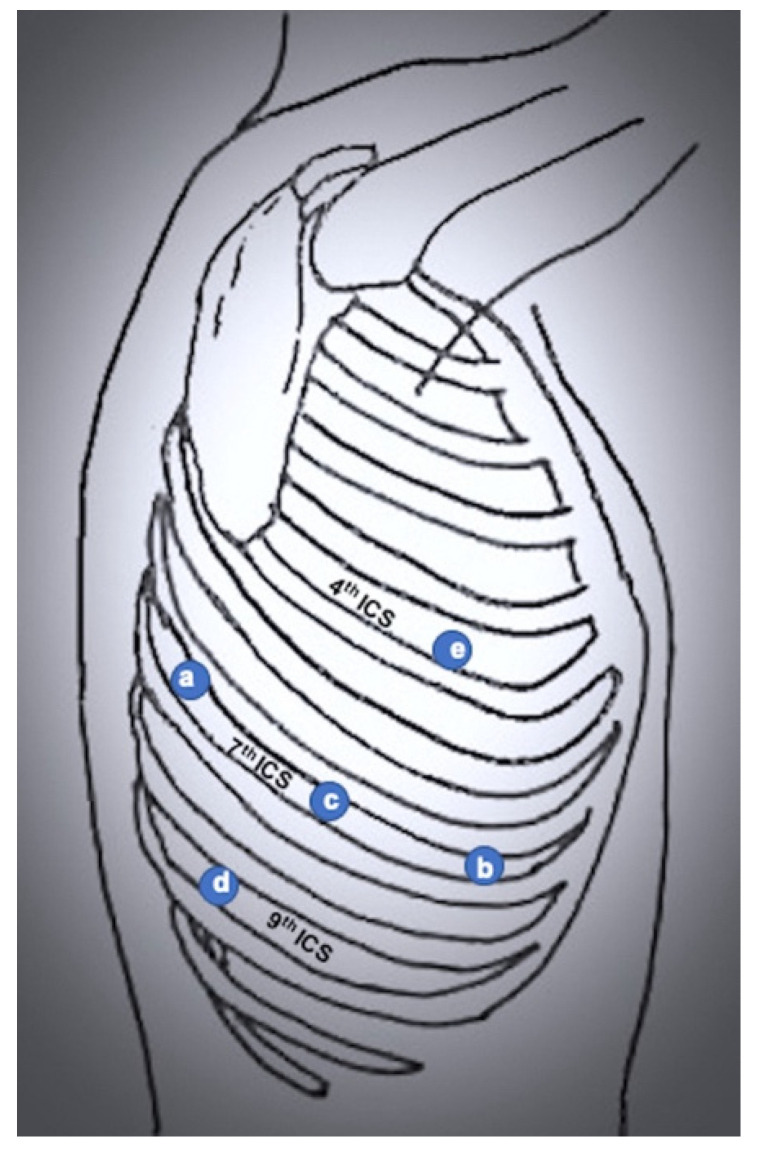
Port mapping of 4-port completely portal robotic lobectomy. (**a**) Robotic left arm 1 (lung retraction); (**b**) robotic left arm 2; (**c**) camera port; (**d**) assistant port; (**e**) robotic right arm.

In 2017, a “five on a dice” port placement was reported [21,22]. This strategy includes a robot port by the tip of the scapula in the 7th intercostal space (tip-up grasper), a port in the 7th intercostal space midaxillary line (bipolar grasper), a camera port in the 7th intercostal space between the first two, a robot port in the 9th intercostal space along the tip of the scapula (Cadiere forceps), and an assistant port in the 4th intercostal space midaxillary line. A specific advantage of this port mapping is that the robotic stapler can be inserted through either the left or the right inferior port with improved control of the vascular staplers during the case (Figure 6).

## 4. Limitations

The main limitation of this paper lies in its nature of narrative review, whose purpose is to identify and summarize a few studies that describe our topic of interest. As it is not a systematic review, no specified protocol was followed, and no critical appraisal or quality assessment tool is available to evaluate its reliability. It should be intended as an overview to guide surgeons in approaching robotic training and the different port placement options. However, it may be unreliable as a source of comprehensive understanding of the state of the art.

## 5. Conclusions

Several port strategies for robot-assisted pulmonary lobectomies have been proposed and described in the literature, each showing its own limitations and advantages. New robotic surgeons may choose their port placement strategy according to personal preference and previous surgical experience, especially regarding open or VATS resections. Robust data comparing different port placements in robotic surgery are lacking. Further research should be directed toward comparisons of clinical outcomes with different robotic approaches.

## Figures and Tables

**Table 1:** Main robotic port strategies and their potential advantages and disadvantages.

	Potential Advantages	Potential Disadvantages
Robot-assisted thoracic surgery (RATS)	Similar to VATS (easier for VATS surgeons)	Dangerous steps performed by the assistant No CO_2_: limited working space compared to completely portal robotic lobectomy
3 or 4 arms ± assistant port [3,5,6]	Similar to VATS	More incisions
Biportal [7]	Similar to VATS Fewer incisions compared to conventional RATS Docking time may be shortened	Retractor used by the bedside assistant Conflicts between instrument and camera may occur
Uniportal [8]	Similar to uniportal VATS Single-incision Docking time may be shortened	Retractor used by the bedside assistant Limited maneuverability (crossed instruments) Experience in uniportal VATS may be needed
Completely Portal Robotic Lobectomy (CPRL)	Smaller incisions CO_2_ insufflation: more working space and easier dissection of tissue planes May be easier if the bedside assistant is a trainee (increased independency of the first surgeon)	Surgeon cannot palpate the lung CO_2_ insufflation may lead to a rise in PaCO_2_ Enlargement of incision for specimen extraction
4 arms + assistant port [15,16]	Caudal port positioning may help in lower lobectomies	Caudal port positioning may impair upper lobe manipulation
4 arms, no assistant port [18]	Fewer incisions compared to conventional RATS	If the assistant is needed for stapling or maneuvering, one instrument may be removed
3 arms + assistant port, 11th intercostal space [19]	Fewer incisions compared to conventional RATS No intercostal access thoracotomy Lobe extraction through a subcostal incision (no rib spreading)	Assistant may be needed for lung retraction May be more challenging in lower lobectomies
“Five on a dice” [21]	Robotic stapler can be inserted through either the left or the right inferior ports	More incisions

## Data Availability

Not applicable (PubMed search).

## Abbreviations

RATS	Robot-Assisted Thoracic Surgery
VATS	Video-Assisted Thoracic Surgery
CPRL	Completely Portal Robotic Lobectomy
